# Functional Characterization of a De Novo *SCN2A* Mixed Variant Linked to Early Infantile Developmental and Epileptic Encephalopathy

**DOI:** 10.1212/NXG.0000000000200344

**Published:** 2026-02-02

**Authors:** Anna Corradi, Antonella Riva, Bruno Sterlini, Lisastella Morinelli, Alessandra Ludovico, Francesca Madia, Pasquale Striano, Martina Albini, Paola Vitale, Michael Pusch, Giulia Lombardo, Maurizio Elia, Nicolas Chatron, Gaetan Lesca, Federico Zara, Raffaele Falsaperla, Loretta Ferrera

**Affiliations:** 1 Department of Experimental Medicine, University of Genoa, Italy;; 2 IRCCS Ospedale Policlinico San Martino, Genoa, Italy;; 3 Department of Neurosciences, Rehabilitation, Ophthalmology, Genetics, Maternal and Child Health, University of Genoa, Italy;; 4 Center for Synaptic Neuroscience and Technology, Istituto Italiano di Tecnologia, Genoa, Italy;; 5 Unit of Medical Genetics, IRCCS Istituto Giannina Gaslini, Genoa, Italy;; 6 Paediatric Neurology and Muscular Diseases Unit, IRCCS Istituto Giannina Gaslini, full member of ERN EpiCARE, Genoa, Italy;; 7 Institute of Biophysics, National Research Council, Palermo, Italy;; 8 Institute of Biophysics, National Research Council, Genoa, Italy;; 9 Neonatal Intensive Care Unit and Neonatal Accompaniment Unit, Azienda Ospedaliero-Universitaria Policlinico “Rodolico-San Marco”, San Marco Hospital, University of Catania, Italy;; 10 Unit of Neurology and Clinical Neurophysiopathology, Oasi Research Institute-IRCCS, Troina, Italy;; 11 Genetics Department, Hospices Civils de Lyon, Lyon, France;; 12 Pathophysiology and Genetics of Neuron and Muscle (PNMG), UCBL, CNRS UMR5261 - INSERM, U1315, Lyon, France; and; 13 Department of Medical Science-Pediatrics, University of Ferrara, Italy.

## Abstract

**Background and Objectives:**

Pathogenic variants in the *SCN2A* gene, encoding the α-subunit type 2 of the voltage-gated sodium channel Na_V_1.2, cause a phenotypic spectrum including 4 major disorders as benign familial infantile seizures, developmental and epileptic encephalopathy, intellectual disability, and autism. Gain-of-function variants resulting phenotypes may be treated with sodium channel blockers, while loss-of-function (LoF) conditions are non-respondent. We focused on the effects of the pathogenic *SCN2A* variant c.4976C>T (p.A1659V) found in heterozygosity in 3 patients affected by DEE non responsive to SCB. We functionally investigated this previously uncharacterized *SCN2A* variant.

**Methods:**

Three individuals with the *SCN2A* c.4976C>T (p.A1659V) variant were studied. This variant was detected by next-generation sequencing (NGS). The nucleotide substitution was inserted by site-directed mutagenesis in a stabilized *SCN2A* plasmid encoding Na_V_1.2. Expression and functional characterization of the Na_V_1.2 A1659V variant was performed in HEK293 cells by western blotting, confocal microscopy, and patch clamp electrophysiology.

**Results:**

The same de novo pathogenic *SCN2A* variant was detected in 3 patients with DEE characterized by early onset, severe ID, and seizures unresponsive to SCB. In 2 patients, the variant is in a mosaic state. The Na_V_1.2 A1659V variant did not affect channel protein expression while exhibiting significant effects on its function as shown by the reduced Na^+^ currents, a shift of the activation curve toward more negative potentials, a shift of the inactivation curve to more negative voltages, and slower kinetics of inactivation compared with native Na_V_1.2 in HEK293 cells. Simulations suggested that the variant increases excitability in neurons.

**Discussion:**

These results revealed the multifaceted functional effect of A1659V variant on channel activity and highlighted the complex genotype-phenotype correlation underlying significant clinical and pharmacological variability in *SCN2A*-related encephalopathies.

## Introduction

The *SCN2A* gene encodes the α- subunit of the voltage-gated sodium channel (Na_V_1.2). The protein is divided into 4 homologous domains (DI-DIV) with 6 transmembrane segments (S1–S6) linked by intracellular and extracellular loops. The voltage sensor domain is made up by the first 4 (S1–S4) transmembrane segments of each domain, whereas the pore domain consists of the S5 and S6 helices. In the conserved loop between domain III (DIII) and domain IV (DIV), a fast inactivation domain has been identified.^1-6^

*SCN2A* is expressed in excitatory neurons and has a critical role in the initiation and propagation of action potentials (APs). During fetal development, Na_V_1.2 is the sole sodium channel expressed at the axon initial segment (AIS), where APs originate.^7,8^ At this stage of life, Na_V_1.2 is crucial for APs generation and propagation.^9^ In later stages of development, Na_V_1.2 is replaced by the orthologous channel Na_V_1.6, encoded by the *SCN8A* gene, which generates APs starting from a lower voltage threshold and is expressed at the level of the nodes of Ranvier and in the distal portion of the AIS. At this point, Na_V_1.2 carries on the role of maintaining APs toward the soma of the neuron.^10,11^ However, during postnatal development, Na_V_1.2 is also expressed in the somatodendritic neuronal compartment, where it takes part in regulating synapses maturation and function, particularly in the prefrontal cortex.^12^

Variants in *SCN2A* may determine changes in the biophysical and electrophysiologic properties of the encoded channel and have been recognized to cause a spectrum of neurodevelopmental disorders (NDs) encompassing different epileptic syndromes (self-limited familial neonatal epilepsy or benign familial infantile seizure (BFIS), developmental and epileptic encephalopathy (DEE), Ohtahara syndrome, epilepsy of infancy with migrating focal seizures, West Syndrome), intellectual disability (ID), and autism spectrum disorder (ASD).^13-15^

Phenotypes can be grouped into 3 categories based on disease severity and outcomes: self-limited conditions, moderately impairing and severe disorders. Self-limited conditions, such as BFIS, involve neonatal seizures, spontaneous remission, and normal neurodevelopment. Moderately impairing disorders, such as ID or ASD, may involve later-onset seizures, though not as a key feature. Severe diseases include epileptic encephalopathies such as West and Lennox-Gastaut syndromes, where frequent seizures significantly disrupt development.^16,17^

From the first discovery in the early 2000's, genotype-phenotype correlations have been proposed for *SCN2A*-related disorders and a rough dichotomy has been created based on the biophysical properties of the altered channel. Gain-of-function (GoF) variants seem more likely associated with early-infantile onset seizures, both self-limited or featuring later mild/moderate developmental impairment. In this case, a good clinical response to sodium channel blockers (SCBs) is expected. Conversely, loss-of-function (LoF) variants have shown to be associated with later-onset epilepsy, with poor response to SCBs,^16^ or nonepileptic phenotypes such as ASD/ID.^5,16-18^

In this study, we functionally investigated a previously uncharacterized *SCN2A* variant (c.4976C>T; p.A1659V) found in 3 patients with early infantile onset developmental and epileptic encephalopathy (EIDEE). The probands showed no clinical response to SCBs. Our study shows that this variant has mixed features that alter the voltage-dependent properties of the channel.

## Methods

### Patient Selection

Patients were ascertained based on clinical suspicion of genetic epilepsy and recruited through a collaboration of third level epilepsy centers, including sites in France and Germany.

### Genetic Analysis

Peripheral blood samples were drawn from the affected individuals and their unaffected parents. Genomic DNA was isolated from 1 mL of blood (Cat.# 69506; Qiagen, Hilden, Germany) and analyzed by next-generation sequencing (NGS) using Epileptic Encephalopathies panel:54 genes (Ion Ampliseq Designer 167 kb, Personal Genome Machine (PGM) platform, software analysis CLC Genomics Workbench and Ion Reporter, eTable 1). The single-nucleotide variant (SNV) identified in *SCN2A* was validated by Sanger sequencing. Primers for coding exon 26 of *SCN2A* gene (26F -5′ TGCTCAACAAACATTGCAGA 3′ and 26R -5′ CCAGCCAGCAGAGGTTGTA 3′) were designed using a Primer3 online tool.^19^ Fibroblasts were obtained from the patients through skin biopsy. The punch was digested to isolate fibroblasts, which were then cultured in Roswell Park Memorial Institute 1640 medium (RPMI) supplemented with 15% fetal bovine serum (FBS), 1% l-glutamine, 100 units/mL penicillin, and 100 μg/mL streptomycin and maintained at 37°C in a 5% CO_2_ humidified atmosphere. DNA was extracted from the fibroblasts and sequenced by Sanger sequencing using the same primers used for blood-derived DNA. PCR products were purified with ExoSAP-it (Cat. # 78201; Thermo Fisher Scientific, Waltham, MA), sequenced on both strands using Big Dye Terminator Cycle Sequencing Kit v3.1 (Cat. # 4337455; Applied Biosystems, Foster City, CA) and resolved on an automated sequencer (ABI 3130xl Genetic Analyzer, Applied Biosystems, Waltham, MA).

### Mutagenesis and Heterologous Expression

Recently, DeKeyser et al. identified in the Na_V_s genes the presence of cryptic bacterial promoter-like elements that determine toxic effects in bacteria cells, hindering DNA manipulation and functional studies. The optimized pIR-CMV-SCN2A-Variant-1-IRES-mScarlet plasmid, a gift from Al George (Addgene plasmid #162279^20^; Research Resource Identifiers (RRID): Addgene162279, Watertown, MA), encoding Na_V_1.2 was used^21^ to introduce the nucleotide substitution of interest in the *SCN2A* gene, by site-directed mutagenesis using the QuikChange Lightning Site-Directed Mutagenesis Kit (Cat. # 210518; Agilent Technologies, Santa Clara, CA) and primers containing the substitution (p.A1659V) in the middle of the sequence designed as follows: forward 5′ GATGATGTCCCTTCCTGTGT GTTTAACATCGGCCTC 3′ and reverse 5′ GAGGCCGATGTTAAACAACACAGGAAGGGACATCATC 3′. All primers were purchased from Eurofins Genomics (Ebersberg, Germany). The correct sequence of the plasmid obtained from mutagenesis was checked by Sanger sequencing. HEK293 cells were cultured in advanced Dulbecco's Modified Eagle Medium (DMEM) supplemented with 10% FBS, 1% l-glutamine, 100 units/mL penicillin, and 100 μg/mL streptomycin and maintained at 37°C in a 5% CO_2_ humidified atmosphere. Cells were transfected with lipofectamine 2000 (Life Technologies, Carlsbad, CA) according to manufacturer's instructions using native or A1659V *SCN2A* plasmids. For electrophysiologic experiments, cells were transfected also with CD8 expressing vector to identify transfected cells.

### Western Blotting

For western blotting analysis, the concentration of protein samples, obtained from HEK293 cells transfected or not with Na_V_1.2 or A1659V constructs, was determined using the Bicinchoninic Acid (BCA) assay and equivalent amounts of proteins were subjected to Sodium Dodecyl Sulfate PolyAcrylamide Gel Electrophoresis (SDS-PAGE) on polyacrylamide gels and blotted onto nitrocellulose membranes (Whatman, St. Louis, MO). Blotted membranes were blocked for 1 hour in 5% milk in Tris-buffered saline (10 mM Tris, 150 mM NaCl, pH 8.0) plus 0.1% Triton X-100 and incubated overnight at 4°C with the anti-PanNav primary antibody (Cat. #S8809; 1:300; Sigma-Aldrich, St. Louis, MO). Membranes were washed and incubated at room temperature (RT) for 1 hour with peroxidase-conjugated secondary anti-mouse antibody (Cat. #1706516; 1:3000; BioRad, Hercules, CA). Bands were revealed with the ElectroChemiLuminescence (ECL) chemiluminescence detection system (ThermoFisher Scientific, Waltham, MA) and imaged by the ChemiDoc Imaging System (BioRad, Hercules, CA). Immunoblots were quantified by densitometric analysis of the fluorograms (Image Lab Software; BioRad, Hercules, CA) obtained in the linear range of the emulsion response.

### Immunocytochemistry, Microscopy, and Image Analysis

HEK293 cells transfected with native or A1659V Na_V_1.2 constructs were fixed in 4% paraformaldehyde for 20 minutes at RT, then washed in Phosphate Buffered Saline (PBS). Cells were subsequently permeabilized with 0.1% Triton X-100 in PBS for 10 minutes at RT and blocked with PBS 3% bovine serum albumin blocking solution for 1 hour at RT. Samples were sequentially incubated with anti-PanNav primary antibody (1:300 diluted in blocking solution) overnight at 4°C. After washes, cells were incubated with the secondary antibody Alexa Fluor 488 (1:500 diluted in blocking solution) for 45 minutes at RT. After washes, coverslips were mounted with Prolong Gold antifade reagent (Invitrogen, Waltham, MA) containing 4′,6′-diamidino-2-phenylindole (DAPI) for nuclear staining. Transfected cells displayed Scarlet signal due to the presence of a Scarlet reporter gene in the pIR-CMV-SCN2A-Variant-1-IRES-mScarlet plasmid. Epifluorescence images at 20X were acquired for the transfection efficiency. Confocal imaging was performed on a Leica TCS SP8 AOBS TANDEM confocal microscope. Confocal scanning was performed with a × 63/1.4 Apochromatic (APO) L W UVI objective using the Leica LAS AF software system with 300 nm between optical sections. ImageJ was used to quantify expression levels by calculating the total fluorescence divided per number of PanNav-positive cells in each field.

### Electrophysiology

Sodium currents were recorded using the patch clamp technique in the whole-cell configuration. Transfected cells were visualized when covered by anti-CD8–coated beads, and recordings were performed at RT. Bath solution contained 145 mM NaCl, 5 mM KCl, 1.8 mM CaCl_2_, 1 mM MgCl_2_, 10 mM Hepes, pH 7.4. Internal solution contained 40 mM CsCl, 10 mM NaCl, 80 mM CsF, 11 mM ethylene glycol tetraacetic acid (EGTA), 10 mM Hepes, 1 mM CaCl_2_, pH 7.3. Series resistance was between 2 and 4 MΩ, and the cell capacitance was between 10 and 25 pF, as measured by the compensating circuit of the amplifier. Data were analyzed with the program Ana^22^ and IgorPro 8.0.4 software (WavemetricsLake, Oswego, OR). Holding potential was −90 mV. Leak and capacitive currents were subtracted using a P/4 protocol. The standard IV protocol was performed by applying 20 ms long pulses to voltages ranging from −60 mV to +90 mV (Δ = 10 mV). Whole-cell conductance (G_Na_) was calculated as $G_{Na}=I/(V-E_{REV})$, where I is the measured peak current, V is the step potential, and E_rev_ is the calculated sodium reversal potential predicted by linear regression of the I–V curve for each cell. To determine the voltage dependence of activation, normalized G_Na_ was plotted against voltage and fitted with the Boltzmann function GGMAX=1+expV−V12/k−1, where V_1/2_ indicates the voltage at half-maximal activation and k is a slope factor describing voltage sensitivity of the channel. To study fast inactivation, we applied a double-pulse protocol: 500 ms long pulses to voltages ranging from −120 mV to 0 mV (Δ = 10 mV) followed by a constant pulse to −10 mV. The fast inactivation steady-state curve was fitted with the Boltzmann function: IIMAX=1+expV−V12/k−1. Activation time constant was obtained from a single exponential fit in response to the applied potential. Inactivation kinetics were assessed by measuring the decay of the current from the peak to 40 ms from the stimulus onset with a single exponential fit. The recovery of fast inactivation protocol was a 2-pulse protocol with varying time intervals with a −10 mV test pulse.

### Computational Modeling

We started from typical optimized models of a rat neocortical or hippocampal CA1 pyramidal cell. The neocortical model, kindly provided by Roy Ben-Shalom, was used as reported in the reference paper,^23^ after replacing both Na_V_1.2 and Na_V_1.6 with the Na_V_1.2 channel here reported. For the hippocampal neuron, we referred to a pre-published model,^24^ substituting the standard literature Hodgkin-Huxley Na^+^ channel with the Na_V_1.2 native kinetics described here, across all compartments and using a 3-dimensional morphologic reconstruction from literature.^25^ In the case of the A1659V substitution, we ran simulations substituting 50% of the native channels with the variant channel. Current-clamp simulations were conducted by injecting increasing current steps to the soma and recording the number of AP from both the soma and AIS. For synaptic stimulation, conducted on the hippocampal model, we colocalized excitatory synapse and inhibitory synapse, both belonging to the standard NEURON class Exp2Syn(),^26^ on the oblique dendrites, and localized following the rationale previously reported.^24^ In NEURON, Exp2Syn is a built-in synapse model representing a double-exponential conductance-based synapse, where the synaptic conductance rises and decays exponentially, allowing the simulation of fast synaptic events with separate time constants for rise and decay. Peak conductances were initially set at 1e-5 nS and 0.5 nS for the inhibitory and excitatory synapses, respectively, with random activation at 40 Hz. We then increased the peak conductance of the excitatory synapses by multiplying it by an increasing factor. All simulations were performed using the NEURON simulation environment.^27^

### Statistical Analysis

Experimental data are expressed as mean ± standard error of the mean (SEM). Normal distribution of data was assessed using the D'Agostino-Pearson normality test. Comparison between mean data were performed using the Student t test, a value of *p* < 0.05 was considered statistically significant. Statistical analysis was performed using Prism (GraphPad Software, Inc.) or Ana and IgorPro 8.0.4 softwares.

### Standard Protocol Approvals, Registrations, and Patient Consents

All methods were performed in accordance with the ethical standards as laid down in the Declaration of Helsinki and its later amendments or comparable ethical standards. Parents/caregivers provided written informed consent for genetic testing, storage, and use of biological samples. The study was conducted following the Helsinki protocol and approved by our local Ethics Committee: Comitato Unico Regionale Regione Liguria n 399/2021.

### Data Availability

The data that support the findings of this study are available in the article. If additional data were required, they might be requested to the corresponding author.

## Results

### Clinical Features and Response to Sodium Channel Blockers

Individual 1 is a boy of 1 year and 1 month born to healthy, non-consanguineous parents. The epilepsy onset was in the first day of life, and seizure semeiology was characterized by limb dystonia, facial dyskinesia, apnea, and tachycardia. The patient was initially treated with phenobarbital, but due to poor seizure control, phenytoin, a nonselective SCB, was added at the intravenous loading dose of 15 mg/kg with no clinical response. Multiple antiseizure medications (ASMs) were tried during time with no benefit, Table 1. Uncontrolled motor and bilateral tonic-clonic seizures (over 50/d) negatively affected neurodevelopment as at the last follow-up (FU, at 1 year and 1 month of age), the boy is not verbal, has absent eye contact, and is unable to roll over or maintain the sitting position (Table 1). Brain MRI at 5 months did not show any anomaly, and his EEG at onset showed a burst suppression pattern, while multifocal epileptiform abnormalities were found at FU.

**Table 1:** Clinical, Instrumental, and Treatment Data of Patients With the c.4976C>T (p.A1659V) Variant

Pt ID, sex (M/F)	1, M	2, F	3, M
Variant inheritance, mosaicism (Y/N)	de novo, Y	de novo, Y	de novo, N
Age at genetic diagnosis (d)	30	NA	6
Age at the study (mo)	13	300	12
Epilepsy onset			
Age (d)	1	3	1
Sz types	Limb dystonia, facial dyskinesia, apnea, tachycardia	FO motor sz	Myoclonic sz, tonic sz, apnea
Epilepsy FU			
Sz types	Tonic spasms, clonic sz, FO motor sz, tonic sz	FO motor sz	Tonic sz
Sz frequency/clusters	>50/d, 15′ clusters	Daily, clusters	>25/d, no clusters
SE (Y/N), age (mo)	Y, 4	Y, UNK	Y, 7
Triggering factors (Y/N)	N	N	N
Sz free (Y/N)	N	N	N
DD/ID (Y/N), degree	Y, severe	Y, severe	Y, severe
Neurologic examination	Averbal, absent eye contact, >muscle tone	Averbal, absent eye contact, quadriparesis, athetoid-dystonic movements, global hypotonia	Averbal, absent eye contact, pyramidal signs, >muscle tone, dystrophia
Other medical conditions	Dysphagia, constipation	NA	Dysphagia, transitory idiopathic SIADH, hip dysplasia
EEG at onset	Burst suppression	Multifocal abnormalities	Burst suppression
EEG at FU (y)	Multifocal epileptiform abnormalities (1)	Slow background, multifocal spikes or SW discharges (3)	Slow background, multifocal epileptiform abnormalities (1)
MRI (mo)	UNR, (5)	Cortical atrophy (12)	Slightly enlarged lateral ventricles, rostral CC T2 signal hyperintensity (12)
ASM: Response	CBZ: nr; PHT: nr; KD: nr; LCM: nr; CZP: nr; CBD: nr; low CS: nr; CLB: pr	VPA: nr; LMT: nr	CBZ: nr (AE: Neutropenia); LCM: nr; LEV: nr; PER: nr; PHT: nr; STP: nr; TPM: nr (AE: Metabolic acidosis); VGB: nr; KD: nr; mexiletine: nr (AE: QT elongation)

Abbreviations: AE = adverse event; ASMs = antiseizure medications; CBD = cannabidiol; CBZ = carbamazepine; CC = corpus callosum; CLB = clobazam; CS = corticosteroids; CZP = clonazepam; DD = developmental delay; F = female; FO = focal-onset; FU = follow-up; ID = intellectual disability; KD = ketogenic diet; LCM = lacosamide; LMT = lamotrigine; LEV = levetiracetam; M = male; N = no; NA = not available; nr = nonresponder; PER = perampanel; PHT = phenytoin; pr = partial responder; Pt = patient; SE = status epilepticus; STP = stiripentol; SW = spike-and-waves; sz = seizures; TPM = topiramate; UNR = unremarkable; UNK = unknown; VGB = vigabatrin; VPA = valproate; Y = yes.

Individual 2 is a woman of 25 years with focal-onset motor seizures from her third day of life. At the time of last FU (25 years), she still experiences daily clusters of focal-onset motor seizures and has severe ID, being not verbal and showing pyramidal signs. Lamotrigine (15 mg/Kg/d) and valproate (20 mg/Kg/d) were tried during the clinical journey but had no control over seizures. Brain MRI performed at 12 months was not contributory. EEG performed at 3 years showed a slow background activity with multifocal spikes and spike waves (Table 1).

Individual 3 is a male patient of 1 year of age showing onset in the first day of life of myoclonic seizures, short tonic seizures, and apnea. The phenytoin was ineffective at onset. Diverse ASMs were tested later-on (Table 1) with no-response. In addition, adverse events were reported: neutropenia caused by carbamazepine (20 mg/kg/d) and QT-elongation provoked by mexiletine. The boy presents severe developmental delay and comorbidities, including transient idiopathic syndrome of inappropriate antidiuretic hormone secretion and hip dysplasia. The MRI at 1 year showed slight enlarged lateral ventricles and rostral corpus callosum T2 signal hyperintensity. The EEG at onset showed burst suppression, persisting at 1 year only during sleep.

**Figure 1:**
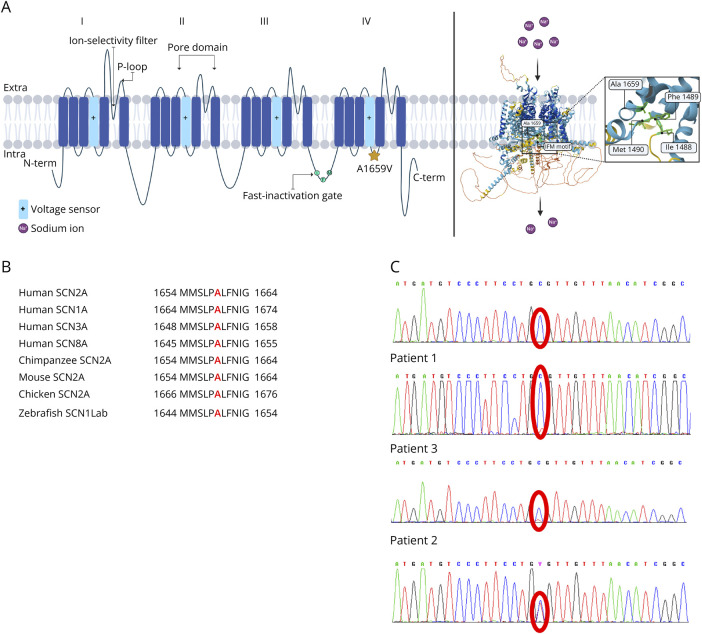
p.A1659V Location in Na_V_1.2 Channel (A) Schematic representation of the topology of the Na_V_1.2 α-subunit showing its 4 homologous domains. Orange star indicates the p.A1659V variant. Right panel shows the AlphaFold structure prediction of the human Na_V_1.2 α-subunit. The A1659 residue and the fast inactivation domain motifs are highlighted in the box on the right. (B) Protein alignment highlights that p.A1659 is conserved in human and in other species Na_V_ α-subunits. (C) Chromatographs of the 3 patients with the same *SCN2A* variant in comparison with a sequence of a healthy control (top panel): central panels show the sequences of patients 1 and 3 (variant in mosaic state), and bottom panel shows the sequence of patient 2: variant in heterozygous state.

NGS revealed the *SCN2A* de novo missense variant c.4976C>T (p.A1659V) in heterozygosity in our probands, indeed the same NGS analysis was performed on their parents, and this variant was not found. No additional genetic variants potentially affecting the phenotype were detected in the probands. The Ala1659 is localized in the intracellular loop between the S4 and S5 segments of the Na_V_1.2 fourth domain (Figure 1A) and is highly conserved both in other human sodium channels and in orthologs of other species (Figure 1B).

Notably, despite similar clinical manifestations (Table 1), we identified the variant in a mosaic state in patients 1 and 3 in blood lymphocytes. In proband 1, the altered allele frequency was 26% (73/279 alleles), while in proband 3, the frequency was 20% (94/462) based on read counts of NGS; the mosaicism was confirmed by Sanger sequencing (Figure 1C). To understand if the variant is also expressed in other tissues, we verified its presence using fibroblast from punch biopsies. For both probands, Sanger sequencing showed the same percentage (26% in patient 1 and 20% in patient 3) of mosaicism in fibroblasts (data not shown).

We used the Shinyapp prediction^28^ tool to evaluate the variant predicted effect on the patients and found this variant predicted to be pathogenic with a probability of 96%.^29^ In addition, the Franklin tool^30^ also indicates p.A1659V as strong pathogenic.^31^

### Characterization of c.4976 C>T (p.A1659V) *SCN2A* Variant

We then investigated changes in the expression profile and biophysical properties of the A1659V Nav1.2 channel. Recombinant cerebral Na_V_s show a high rate of recombination when propagated in bacteria, hindering DNA manipulation and functional studies. We therefore used the stabilized pIR-CMV-SCN2A plasmid encoding Na_V_1.2 to insert the pathogenic variant by site-directed mutagenesis to overcome these technical difficulties.

### A1659V Variant Does Not Affect Na_V_1.2 Channel Protein Expression

**Figure 2:**
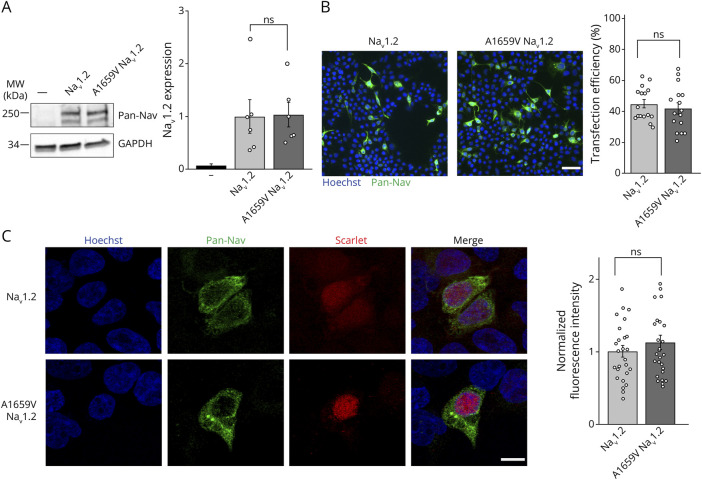
A1659V Variant Does Not Affect Na_V_1.2 Channel Expression (A) Protein extracts from HEK293 cells transfected with either native or A1659V Na_V_1.2 or untransfected (indicated with a horizontal bar) were analyzed by western blotting using anti-PanNav antibody. *Left:* Representative immunoblot. *Right*: Densitometric analysis of the immunoreactive bands. GAPDH was used as loading control. The expression levels were normalized on native Na_V_1.2 mean expression value and are shown as means ± SEM of n = 6 independent experiments. (B) Representative images of HEK293 cells transfected with either native or A1659V Na_V_1.2 and immunolabelled with anti-PanNav antibody (green). DAPI staining was used to visualize nuclei (blue). Scale bar, 50 μm. *Right*: The percentage of transfection efficiency was calculated as the ratio between the anti-PanNavpositive cells and total cells in the same field. Data represent means ± SEM with superimposed individual values from n = 3 independent experiments. (C) *Left*: representative confocal images of HEK293 cells transfected with either native or A1659V Na_V_1.2 and immunolabelled with anti-PanNav antibody (green). DAPI staining was used to visualize nuclei (blue), and Scarlet signal is indicative of transfection (red). Scale bar, 5 μm. *Right*: Quantification of PanNav fluorescence intensity calculated as the ratio between the total PanNav fluorescence signal and the number of PanNav-positive cells in the same field. The fluorescence intensity levels were normalized on native Na_V_1.2 mean intensity value and are shown as means ± SEM with superimposed individual values from n = 3 independent experiments. Statistical analysis was performed using the unpaired Student *t* test.

We assessed the effect of the variant on channel protein expression. To this aim, the cDNA of native and A1659V Na_V_1.2 were transfected in naïve HEK293 cells, and the protein extracts were analyzed by western blot (Figure 2A, left, eFigure_1 shows the full western blot image). The A1659V Na_V_1.2 showed no significant differences in expression levels compared with the native Na_V_1.2 (Figure 2A, right). We also verified the absence of endogenous expression of Na_V_1.2 in HEK293 cells (Figure 2A, left first lane). Transfected HEK293 were also analyzed by immunofluorescence using anti-PanNav antibody (Figure 2, B and C, left). No differences in the number of transfected cells (Figure 2B, right) were identified between native and A1659V Na_V_1.2. Moreover, image analysis by confocal microscopy did not display any differences in the signal distribution nor in the fluorescence intensity quantification of cells transfected with native or A1659V Na_V_1.2 (Figure 2C, right). We conclude that the A1659V variant does not affect the expression level of Na_V_1.2 channel.

### A1659V Variant Modifies Na_V_1.2 Channel Activity and Biophysical Properties

**Figure 3:**
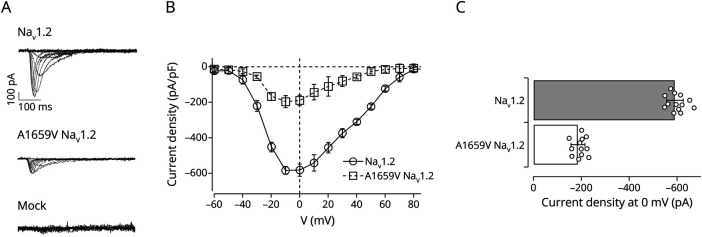
A1659 Variant Exhibits a Smaller Na + Current About the Na_V_1.2 Native Channel (A) Whole-cell sodium representative current traces recorded from cells transiently expressing native (top panel), or A1659V (central panel) Na_V_1.2 or only CD8 plasmid (mock, bottom panel). Currents were elicited from a holding potential of −90 mV with 20 ms depolarizing voltage steps between −60 and +90 mV. (B) Average current density-voltage relationships for HEK293 cells transfected with native (dot symbols and solid lines) or A1659V Na_V_1.2 (square symbols and dashed lines) as indicated. (C) Mean of density current values measured at 0 mV (I_peak_) for indicated channels (native Na_V_1.2, filled bar; A1659V Na_V_1.2, striped bar). Data are expressed as mean ± SEM from n ≥ 12 independent experiments.

We investigated functional properties of A1659V variant by the patch-clamp technique in whole-cell configuration in transiently transfected HEK293 cells with either native or the A1659V Na_V_1.2 variant. We observed a strong reduction of inward sodium currents in cells transfected with the A1659V channel (Figure 3A): the A1659V Na_V_1.2 exhibited a significant reduction of current density of about 80% about the reference in response to depolarizing stimuli that activate the voltage-gated sodium channel (Figure 3, B and C).

**Figure 4:**
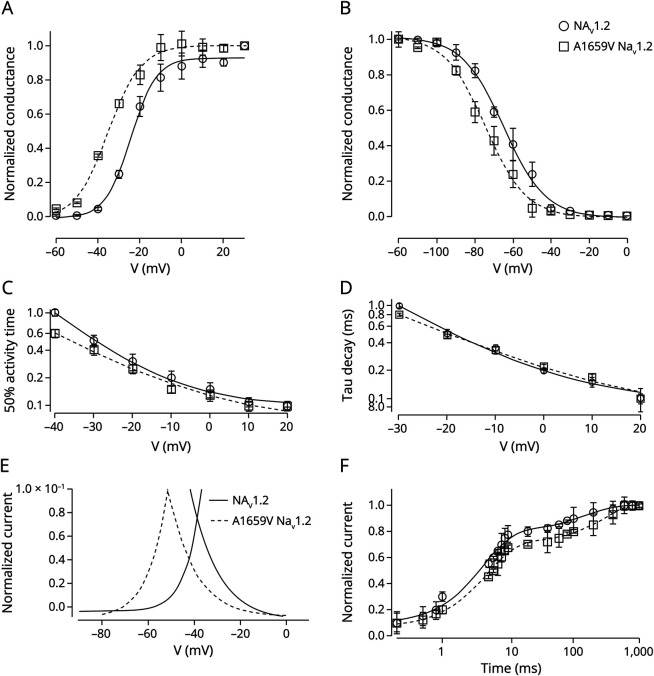
A1659 Variant Shows Altered NaV1.2 Biophysical Properties (A) Superimposed voltage-dependence curves of steady-state activation. (B) Superimposed voltage-dependence curves of steady-state inactivation. (C) Time to half-activation at the indicated membrane potentials. (D) Inactivation time constants derived from current decay during depolarization at the specified voltages. (E) Window currents for native and A1659V channels, obtained by superimposing the normalized activation and inactivation curves. (F) Time course of recovery from fast inactivation, fitted using a double-exponential function. In all panels, recordings were from cells expressing either native or A1659V Na_V_1.2 channels, dot symbols (mean ± SEM) and solid lines (corresponding fit) represent data from the reference channel, while square symbols (mean ± SEM) and dashed lines (corresponding fit) correspond to the data of the A1659V Na_V_1.2. Quantitative data for all measured parameters are summarized in Table 2. Data are presented as mean ± SEM from n ≥ 12 independent experiments.

In addition, we observed that the A1659V variant showed significant changes in the properties of voltage dependence of activation and inactivation: we observed a significant hyperpolarizing shift of about −10 mV in the activation curve (Figure 4A and Table 2) and a hyperpolarizing shift of about −10 mV of the voltage of half inactivation (Figure 4B and Table 2). Activation and inactivation kinetics of the native and A1659V channels were similar at membrane potentials governing channel gating (Figure 4, C and D). The analysis of window currents, derived from the superimposition activation and inactivation curves, revealed no significant differences of the area of the window current but a significant left-shift of the A1659V window currents compared with native protein (Figure 4E). Furthermore, comparison of the kinetics of recovery from inactivation between the reference and A1659V Na_V_1.2 revealed that the fast time constant (τ_1_) of the variant was larger than that of the native channel (Figure 4F). Therefore, the biophysical characterization of A1659V variant revealed complex functional properties of the A1659V Na_V_1.2 channel. Specifically, while the voltage dependence of activation suggested a GoF effect, both the voltage dependence and kinetics of inactivation were consistent with a LoF mechanism, as evidenced by a significantly larger recovery time constant (τ1) from fast inactivation in the A1659T variant compared with the native protein (Table 2).

**Table 2:** Functional Features of Na_V_1.2 and A1659V Channels

Channel	Voltage dependence of activation		Voltage dependence of inactivation		Time of half-activation (ms)	Time constant of inactivation (ms)	Recovery from fast inactivation			
	V_1/2_ (mV)	k (mV)	V_1/2_ (mV)	k (mV)			τ_1_ (ms)	A_1_	τ_2_ (ms)	A_2_
Na_V_1.2	−24.04 ± 0.77	6.8 ± 0.1	−64.77 ± 0.83	6.0 ± 0.8	0.77 ± 0.003	0.54 ± 0.006	0.33 ± 0.05	0.79 ± 0.08	23.61 ± 2.63	0.21 ± 0.05
Na_V_1.2 A1659V	−35.10 ± 0.20^a^	7.8 ± 0.3	−74.51 ± 1.23^a^	6.8 ± 1.11	0.54 ± 0.006^a^	0.52 ± 0.005	0.65 ± 0.24^a^	0.64 ± 0.05	20.98 ± 1.73	0.36 ± 0.06

V_1/2_ is voltage of half maximal activation or inactivation as indicated. K is the slope factor and describes voltage sensitivity of the channel. Time constant of half-activation and time constant of inactivation indicate the time dependence of biophysical properties of both Na_V_1.2 and Na_V_1.2 A1659V channel. τ_1_, A_1_, τ_2_, and A_2_ are the time constants and amplitudes of the recovery of fast inactivation fitted with a double exponential. Data are expressed as mean ± SEM from n ≥ 12 independent experiments.

a *p* < 0.01 vs WT determined by the Student test.

### Computational Modeling in Neocortical and Hippocampal Rodent Pyramidal Cells

**Figure 5:**
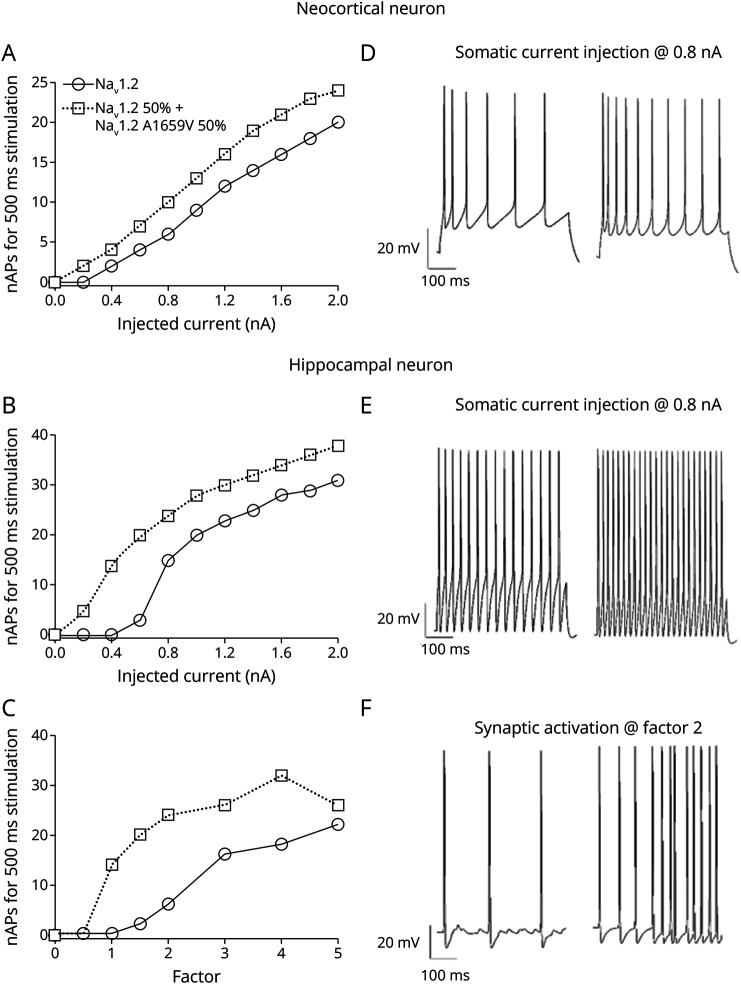
Simulated Neuronal Excitability in a Mouse Cortical and a Rat Hippocampal Pyramidal Cell The cell expresses either native Na_V_1.2 (circular symbol and solid line) or a combination of Na_V_1.2 and the Na_V_1.2 A1659V variant, each contributing 50% to the total channel density (square symbol and dotted line). Number of spikes as a function of the current injected through somatic stimulation for 500 ms, in a (A) neocortical neuron and (B) hippocampal neuron; (C) number of spikes in response to 40 Hz synaptic stimulation for 500 ms in a hippocampal neuron. Example of modeled traces in response to 0.8 nA current injections in a (D) neocortical neuron and (E) hippocampal neuron (left Na_V_1.2 and right Na_V_1.2 A1659V); (F) example of modeled traces under synaptic stimulation with a doubling of the weight of the netcon linking the excitatory synapses to the soma (left Na_V_1.2 and right Na_V_1.2 A1659V).

To gain insights into the effect of the variant on single-cell firing behavior, we decided to test the kinetics of both native and A1659V Na_v_1.2 (reported in Figure 4) by computational modelling. We used 2 previously published models of neocortical and hippocampal rat pyramidal cells as templates^23,24^ for neocortical and hippocampal neuron, respectively, replacing the standard Hodgkin-Huxley Na^+^ channels, originally inserted, with the Na_V_1.2 model described in this study. After configuring the native cell model, we substituted half of the healthy channels with the A1659V variant. Figure 5, A–C clearly shows that replacing 50% of the channels with the variant leads to increased firing frequency and a lowered AP threshold in all simulated cells. This effect is especially pronounced in hippocampal neurons where the minimum current required to evoke an AP decreases from 0.6 nA to 0.2 nA, and at 0.6 nA the number of APs triples. This phenomenon is illustrated by the example traces in Figure 5, D, E, and F, where both neocortical and hippocampal neurons, under current injection and synaptic activation, show a narrower interspike interval along the AP train in the presence of the Na_V_1.2 variant.

While the computational analysis unambiguously demonstrates a GoF effect, manifested as increased excitability and reduced firing threshold, the functional study of the A1659V variant reveals that this substitution alters the channel's biophysical properties, producing both GoF and LoF effects compared with the reference protein. Together, these 2 approaches support the hypothesis that the variant exhibits mixed functional properties.

## Discussion

The 4 homologous neuronal sodium channels (Na_V_1.1, 1.2, 1.3, and 1.6) are essential for neuronal firing and brain development.^32,33^ Their expression and localization vary across brain regions. Na_V_1.2 is primarily found at AISs and nodes of Ranvier in myelinated fibers during early development, later shifting to unmyelinated AIS and dendrites in adults.^11^ Consequently, changes in Na_V_1.2 biophysical properties can result in diverse NDs.

Within phenotypic heterogeneity, grouping based on genetic/functional features has also been attempted.^17^ Missense GoF variants commonly lead to early-onset seizures (median age at onset = 17 days), both self-limited and with later delayed psychomotor development. Conversely, missense LoF variants are associated with later-onset epilepsies and nonepilepsy phenotypes such as ASD/ID.^34^

From an electrophysiologic perspective, some variants fail to be strictly classified into GoF or LoF. Indeed, this dichotomy appears to be less rigid, as several *SCN2A* variants exhibiting complex biophysical properties and have been classified as “mixed” and identified in patients with epilepsy characterized by neonatal seizures.^35-37^

In this study, we investigated the biophysical properties of a previously uncharacterized Na_V_1.2 missense variant leading to a severe, encephalopathic phenotype in the affected patients. Peculiarly, the variant reached clinical attention due to the unexpected treatment response. All participants had clinical features compatible with a GoF variant but showed no clinical response to SCB. When characterizing the in vitro properties of the c.4976C>T (p.A1659V) variant, we observed no effect on the Na_V_1.2 channel protein expression levels; hence, our missense variant does not influence the protein synthesis and maturation processes. Then, we measured the current passing through the altered channel and observed smaller currents in the A1659V variant compared with native protein. Based on this, we hypothesized that the variant caused a LoF. However, the variant also altered the voltage dependence of activation and inactivation. The hyperpolarizing shift of the activation curve is in principle a GoF effect, whereas the leftward shift of the inactivation curve represents a LoF. In a similar study, Miao et al. analyzed 2 de novo *SCN2A* variants: p.P1658S (adjacent to p.A1659V) and p.A1773T in the S6 segment of domain IV. They demonstrated that both missense variants were either LoF (P1658S) or mixed (A1773T). Specifically, P1658S caused a complete loss of detectable currents, while A1773T resulted in a significant reduction in current density, accompanied by a hyperpolarizing shift of the voltage dependence of activation and a hyperpolarizing shift in fast inactivation. Therefore, the functional profile of the A1773T variant closely resembled the electrophysiologic alterations observed for the A1659V variant, suggesting a potential functional correlation between these residues.^35^

To better understand the association between the A1659V variant and the patients' phenotype, we used a computational model based on rodent neurons. Models expressing both the native channel and the A1659V Na_V_1.2 variant exhibited a higher firing frequency and a lower spiking threshold compared with the reference channel alone, across the full range of injected currents and under more physiologic conditions achieved through synaptic stimulation. These results suggest that, in these systems, the variant is associated with a GoF effect. This effect was also consistent, across all tested configurations, with the increased neuronal excitability previously observed for the M1770L variant using a similar modeling strategy.^38^

We assume that the substitution of A1659V could change the conformation of the S4-S5 linker of DIV (Figure 1), a region of the channel that regulates the Na_V_1.2 fast inactivation mechanism.^35,39^ Noteworthy both the P1658S and A1773T variants, as well as the A1659V, map to the channel sequences involved in fast inactivation processes,^40^ causing decreased currents and LoF or mixed-associated phenotypes. Clinically, Miao variants exhibited LoF or mixed properties,^35^ including reduced current density and enhanced fast inactivation. However, patients with these variants displayed milder phenotypes compared with the severe cases observed in our cohort. The mean age at seizures onset was 7 months, and the effect on development was moderate or absent. All our 3 patients had severe developmental delay/ID. These findings suggest that even pathogenic variants in adjacent aminoacidic residues may lead to fine differences in the biophysical channel properties with diverse phenotypic expression.

Our probands do not respond to SCB, like other patients carrying mixed-effects variants (A1773T, R1312T, and E1211K), with electrophysiologic properties similar to those identified for the A1659V variant here described. In vitro application of SCB to these variants results in a further reduction of channel function, thereby explaining the lack of treatment efficacy in these patients. We hypothesize that in the case of the A1659V variant as well, these compounds may fail to produce a beneficial effect.^35,37,41^ Furthermore, Garcia et al. recently demonstrated, in vitro and *in vivo*, that the specific blockade of the Na_V_1.2 channel paradoxically induces neuronal hyperexcitability. Based on this finding, we hypothesize that LoF variants could also lead to an epileptic phenotype in pediatric patients and it may also account for the lack of efficacy of SCB in epileptic patients carrying specific Na_V_1.2 variants, as observed in the probands described here. The reduced channel activity, resulting from LoF or mixed variants, likely underlies the pharmacoresistance.^23^ Based on the clinical pharmacoresistance to SCB and the mixed functional effects observed in vitro, alternative treatment strategies may be considered. Given the presence of GoF features such as enhanced neuronal excitability in computational models, agents acting on excitatory-inhibitory balance (e.g., GABAergic modulators) or non-sodium channel targeted therapies (e.g., cannabidiol, steroids, or mTOR pathway modulators) might offer therapeutic benefit in selected cases. However, the variable phenotype underscores the importance of personalized approaches and the need for further functional testing to guide treatment selection.

Furthermore, the strong functional effect of the A1659V variant on channel function is further supported by the severity of the resulting epileptic phenotype in our cohort, and the full clinical expression of the nucleotide substitution at the mosaic state. Indeed, approximately 20% of A1659V cells in 2 analyzed tissues (lymphocytes and fibroblasts) appear to be sufficient to drive the phenotypic expression in 2 patients (pts #1 and #2). Of course, the exact percentage of mosaicism in the brain cannot be inferred based on the results on peripheral tissues. Further studies, with iPSC-derived neurons, could shed light on the possibility that reduced percentage of neurons can cause impaired network dynamics and determine severe clinical manifestation, highlighting the importance of the A1659 residue for the physiologic Na_V_1.2 channel function.

Our results highlight the significant physio-pathologic effect of the A1659V variant on neuronal function and provide the functional basis of the severe phenotype observed in our cohort of patients. Moreover, we unhinge the conventional dichotomy between LoF and GoF variants in genotype-phenotype correlation^17,42-44^ and provide hints for early functional testing in the clinical practice, to guide treatment approaches and potentially affect the neurodevelopmental trajectories of patients with *SCN2A*-related disorders.

## Glossary

AIS: axon initial segment
AP: action potential
ASD: autism spectrum disorder
ASM: antiseizure medication
BFIS: benign familial infantile seizure
DEE: developmental and epileptic encephalopathy
FBS: fetal bovine serum
G_Na_: whole-cell sodium conductance
ID: intellectual disability
NGS: next-generation sequencing
RT: room temperature
SCB: sodium channel blocker
